# Staging FDG PET/CT prediction of bone marrow involvement by diffuse large B-cell lymphoma leading to delayed recognition of multiple myeloma

**DOI:** 10.1007/s12308-025-00667-1

**Published:** 2025-11-04

**Authors:** Amanda C. Gibbs, Prem P. Batchala, Chelsea E. Gottlieb, Bryan O. Balle, Ifeyinwa E. Obiorah, Laahn H. Foster, Emily C. Ayers, Jeffrey W. Craig

**Affiliations:** 1 Department of Pathology, University of Virginia Health System (UVA Health), 1215 Lee Street, PO Box 800904, Charlottesville, VA 22908-0904 USA; 2 Department of Radiology and Medical Imaging, UVA Health, Charlottesville, VA USA; 3 Charlottesville Pathology Associates, Charlottesville, VA USA; 4 Department of Medicine – Hematology/Oncology, UVA Health, Charlottesville, VA USA

**Keywords:** Diffuse large B-cell lymphoma, Plasma cell myeloma/multiple myeloma, Positron emission tomography/computed tomography, Bone marrow, Staging

## Abstract

**Background:**

18-Fluorodeoxyglucose positron emission tomography/computed tomography (FDG PET/CT) scans have largely replaced bone marrow biopsy in the staging workup for diffuse large B-cell lymphoma (DLBCL).

**Purpose/Methods:**

Here, we highlight a pitfall of FDG PET/CT for assessing bone marrow involvement, detailing the clinical course of a patient diagnosed with otherwise limited-stage DLBCL who exhibited diffuse bone marrow FDG uptake, felt to be compatible with extensive lymphomatous infiltration. In accordance with current National Comprehensive Cancer Network guidelines, this finding circumvented the need for a formal staging bone marrow biopsy procedure.

**Results:**

Subsequent clinical developments revealed extensive bone marrow involvement by a second, unexpected malignancy (multiple myeloma), casting doubt on the assumption that the initial diffuse marrow uptake was attributable to DLBCL.

**Conclusion:**

The presented case emphasizes the challenges of interpreting diffuse FDG uptake within the bone marrow compartment and highlights the inherent contribution of histomorphology for the accurate diagnosis and classification of hematolymphoid neoplasms.

## Introduction

Although a small percentage of patients with diffuse large B-cell lymphoma (DLBCL) are found to have single/isolated lesions, the vast majority present with more widespread disease [1]. Clinical staging has traditionally relied on the Ann Arbor classification system, which stratifies lymphoma based on the number and location of involved lymph node groups and the presence of extranodal manifestations [2]. Prior to the widespread availability of 18-fluorodeoxyglucose positron emission tomography/computed tomography (FDG PET/CT) scans, a cornerstone of this staging process had been the bone marrow biopsy, historically integral for determining the type (concordant vs. discordant) and extent of marrow involvement [3, 4]. Bone marrow infiltration has been documented in approximately 15–20% of DLBCL cases [5], with concordant histology (i.e., high-grade/large cell) being an independent predictor of poor clinical outcome [6].

Over the past decade, FDG PET/CT has had a significant impact on the staging and management of many lymphomas [7]. Early studies investigating the use of FDG PET/CT for detecting DLBCL in bone marrow showed impressive sensitivity (88.7%) and specificity (99.8%) [8], prompting the Lugano classification authors to eliminate the requirement for bone marrow biopsy in patients with positive FDG PET/CT as well as in patients with negative FDG PET/CT whose management would not change based on the presence of discordant (i.e., low-grade) lymphoma [9].

## Clinical history

**Fig. 1:**
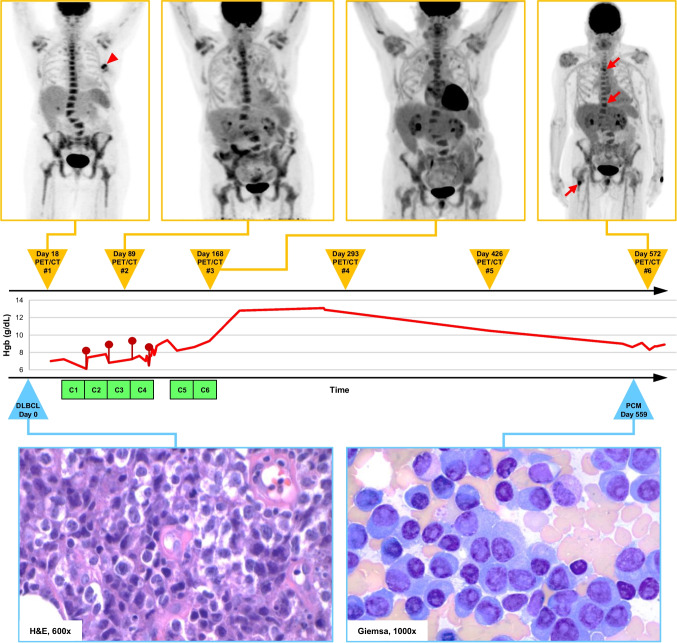
Timeline highlighting the temporal relationships between major clinical events including the biopsy procedures leading to the diagnoses of diffuse large B-cell lymphoma (DLBCL, day 0) and plasma cell myeloma (PCM, day 559), as well as all FDG PET/CT scans (#1–6) and cycles of chemotherapy (C1-C6). The patient’s hemoglobin level (red trendline) is provided for context, with maroon lollipops corresponding to the transfusion of 1–2 units of packed red blood cells. Selected whole body FDG PET/CT maximum intensity projection images (top row) demonstrate persistence of the axial and appendicular skeletal uptake first appreciated at the time of initial DLBCL staging. In addition to the diffuse uptake, more intense discrete foci are noted in the most recent PET/CT (upper right panel) in the spine and right femur (red arrows). Note the FDG-avid left breast mass in the upper left panel (red arrowhead), corresponding to the only biopsy proven site of lymphoma, completely resolved in the subsequent PET/CT. The patient’s H&E-stained left breast core biopsy (bottom left panel, original magnification ×600) contains a diffuse infiltrate of large pleomorphic lymphocytes with high mitotic rate and evolving tumor necrosis, consistent with DLBCL. Immunohistochemical studies (not shown) revealed a sheet-like proliferation of large CD20-positive B cells with co-expression of CD45, BCL6, and MUM1, but no significant reactivity for CD10, CD30, or BCL2. Her subsequent Giemsa-stained bone marrow aspirate smear (bottom right panel, original magnification ×1000) features a dense proliferation of atypical plasma cells with irregular nuclei and prominent nucleoli, supporting a diagnosis of PCM

A 72-year-old female with a past medical history notable for treated hepatitis C infection initially presented to an outside hospital with a non-tender left breast lump. Her physical exam was otherwise unremarkable. Diagnostic mammography and ultrasound identified an ill-defined, 4.0 × 2.1 cm hypoechoic mass in the left breast, classified as BI-RADS Category 5. Core needle biopsy revealed DLBCL, non-germinal center B-cell type per Hans algorithm (Fig. 1). Additional analyses such as in situ hybridization for Epstein Barr virus RNA and fluorescence in situ hybridization for *MYC*, *BCL2*, and *BCL6* rearrangements could not be performed due to the exhaustion of available tissue.

Staging with FDG PET/CT demonstrated an FDG-avid left breast lesion (SUV max = 6.8) as well as diffuse hypermetabolic activity throughout the axial and appendicular skeleton, deemed compatible with bone marrow infiltration by lymphoma (Fig. 1). There was no hypermetabolic lymphadenopathy or evidence of other involved extranodal sites. Based on these findings, the patient’s disease was classified as Stage IV DLBCL, and she was placed in the high-intermediate risk group (IPI 3) [10]. Her labs at the time of diagnosis were notable for pancytopenia, which was attributed to lymphomatous infiltration of her marrow cavity; her calcium and creatinine levels were normal. Protein electrophoresis and free light chain studies were not performed at this time.

The patient’s first cycle of immunochemotherapy consisted of standard R-CHOP (rituximab plus cyclophosphamide, doxorubicin, vincristine, and prednisone) and led to a significant clinical reduction in the size of her left breast mass. Her subsequent three immunochemotherapy cycles were adjusted to Pola-R-CHP (polatuzumab vedotin and rituximab plus cyclophosphamide, doxorubicin, and prednisone) [11] (Fig. 1). She experienced ongoing anemia throughout therapy, necessitating multiple packed red blood cell transfusions.

Interim FDG PET/CT after three total cycles of immunochemotherapy showed near-complete resolution of her left breast lesion (Deauville 2) (Fig. 1). There was, however, persistent diffuse bone marrow uptake. The patient went on to complete six cycles of chemotherapy, with the final two cycles converted to CHOP due to concern for drug-induced pneumonitis. End-of-treatment (EOT) FDG PET/CT demonstrated no evidence of residual/recurrent lymphoma in the left breast (Fig. 1). However, diffuse bone marrow uptake persisted, remaining largely unchanged from the initial imaging. In context, the persistent marrow uptake seen in both interim and EOT FDG PET/CT studies was felt to represent physiological marrow reconstitution following therapy, leading to a determination of complete response at the latter time point. However, persistent hematologic malignancy of either concordant or discordant histology remained a plausible alternative. Following a brief recovery period, the patient’s anemia unexpectedly recurred shortly thereafter (Fig. 1).

Her peripheral counts continued to worsen over several months following her EOT FDG PET/CT. A bone marrow biopsy was ultimately performed approximately 13 months after the completion of her DLBCL-directed therapy, in the setting of worsening anemia, revealing marked hypercellularity and extensive infiltration by a lambda-restricted plasma cell neoplasm accounting for 88% of the total marrow cellularity, in keeping with plasma cell myeloma/multiple myeloma (Fig. 1). Background trilineage hematopoiesis was markedly suppressed, and there was no evidence of lymphoma. Cytogenetic testing revealed an abnormal hyperdiploid profile (Karyotype: 53,X,-X,+3,+5,+7,+9,+11,+15,+add(19)(q13.4), + 21[11]/46,XX[9]). Her most recent PET/CT from that time showed symmetric mildly heterogeneous radiotracer uptake within the axial and proximal appendicular skeleton (Fig. 1).

## Discussion

Our patient’s multiple myeloma diagnosis was rendered approximately 18 months after her DLBCL was first discovered and her initial staging FDG PET/CT demonstrated diffuse axial and appendicular uptake. By the time a bone marrow biopsy was performed, her marrow cavity was completely replaced with neoplastic plasma cells despite having completed full induction therapy for lymphoma just a year earlier. She was also noted to be pancytopenic at the time of her DLBCL diagnosis, and her anemia persisted throughout most of her therapy despite prompt resolution of the only biopsy-proven site of lymphoma. Additionally, her peripheral counts began to decline just a few months after her treatment ended, in the setting of continuously abnormal bone marrow FDG uptake. Taken together, these findings retrospectively support the presence of a plasma cell neoplasm that was not fully recognized in the setting of newly diagnosed lymphoma but likely accounted for all the patient’s clinical and laboratory abnormalities, save her unrelated left breast mass. This case highlights the potential for negative health consequences stemming from the over-reliance on FDG PET/CT for staging of newly diagnosed DLBCL, as the initial decision to omit the bone marrow biopsy, in accordance with National Comprehensive Cancer Network guidelines [12], likely led to a delay in the diagnosis of concurrent multiple myeloma. However, we acknowledge that definitive proof of this presumption would have required earlier bone marrow sampling and/or other components of an initial myeloma-directed workup.

A recent systematic review indicates that the anticipated (median) sensitivity (77.40%) and specificity (91.65%) of FDG PET/CT for detecting bone marrow involvement by DLBCL are likely lower than initially appreciated [13]. Also troubling is the sub-par positive predictive value of 63.60% (median value among published studies), indicating that FDG uptake is frequently identified in the absence of biopsy-confirmed bone marrow involvement. This problem may be exacerbated in the setting of diffuse bone marrow signal, which has been defined by some as homogeneous FDG uptake in the axial and appendicular skeleton higher than liver uptake or as heterogeneous uptake regardless of intensity [14]. A diffuse pattern of bone marrow uptake is present in a subset of DLBCL cases, including 5.5% with a purely diffuse pattern and 18.1% with a mixed (diffuse and focal) pattern [15]. However, diffuse marrow uptake is considered less specific for lymphomatous infiltration than focal marrow uptake in the context of DLBCL staging and can therefore be prone to misinterpretation [16, 17]. Meanwhile, diffuse marrow FDG uptake is comparatively more common in cases of plasma cell myeloma [18], occurring either alone or in combination with focal uptake, and has been associated with elevated bone marrow plasma cell infiltration rates, less differentiated plasma cell morphology, and multiple adverse prognostic markers [19, 20]. The majority of false-positive FDG PET/CT results in the setting of diffuse marrow uptake have been attributed to reactive, inflammatory, or other non-malignant conditions [17, 21, 22], particularly diffuse hematopoietic marrow hyperplasia (i.e., red marrow reconversion) such as may be seen during bone marrow recovery following chemotherapy. Our case is especially noteworthy in this regard, as it represents an example where FDG PET/CT findings, initially attributed to lymphoma, complicated the diagnosis of a second malignancy. We are unaware of any similar reports within the existing medical literature.

The attribution of the initial FDG PET/CT findings to DLBCL in this case appears to have had several relevant consequences, including (1) the patient’s disease being upstaged from stage IE to stage IV, (2) the patient’s international prognostic index (IPI) score increasing by one point to 3 (high-intermediate), and (3) the patient’s cytopenias being attributed to lymphoma and not further investigated, potentially contributing to the need for multiple blood transfusions. Although our patient did not suffer any major pathological fractures prior to her myeloma diagnosis, it is reasonable to be fearful of such an outcome given the significant osteolytic potential of plasma cell neoplasms. At the time of this report, our patient is over a year out from initiating dara-RVD (daratumumab plus lenalidomide, bortezomib, and dexamethasone) therapy that has since been switched to daratumumab-velcade maintenance due to cost barriers and cytopenias associated with lenalidomide. She has maintained a very good partial response but was deemed ineligible for stem cell transplant. Her DLBCL remains in remission.

In summary, it is in the best interest of patients and the medical community to interpret FDG PET/CT findings with a high index of clinical suspicion when imaging is potentially incongruent with clinical and other laboratory data. To this end, we recognize Alyamany et al. who propose that diffuse marrow uptake not be used as justification to avoid formal staging bone marrow biopsy, even in the presence of clinical indicators associated with elevated pretest probability (e.g., cytopenias, bulky disease, elevated LDH) [23].

## Data Availability

All relevant data are included in the report.
